# Genetic and cytometric analyses of subcutaneous adipose tissue in patients with hemophilia and HIV-associated lipodystrophy

**DOI:** 10.1186/s12981-022-00432-9

**Published:** 2022-03-04

**Authors:** Takanobu Mashiko, Kunihisa Tsukada, Hitomi Takada, Szu-Hsien Wu, Koji Kanayama, Rintaro Asahi, Masanori Mori, Akira Kurisaki, Shinichi Oka, Kotaro Yoshimura

**Affiliations:** 1 Department of Plastic Surgery, Jichi Medical University, 3311-1, Yakushiji, Shimotsuke-Shi, Tochigi 329-0498 Japan; 2 Department of Plastic Surgery, University of Tokyo, 7-3-1, Hongo, Bunkyo-Ku, Tokyo, 113-8655 Japan; 3 AIDS Clinical Center, National Center for Global Health and Medicine, 1-21-1, Toyama, Shinjuku-ku, Tokyo, 162-8655 Japan; 4 Stem Cell Technologies Laboratory, Graduate School of Biological Sciences, Nara Institute of Science and Technology, 8916-5, Takayama, Ikoma, Nara 630-0192 Japan

**Keywords:** Human immunodeficiency virus, HIV-associated lipodystrophy, Lipoatrophy, Gene analysis, Adipose-derived stem cell, Macrophage

## Abstract

**Background:**

The authors recently performed plastic surgeries for a small number of patients with hemophilia, HIV infection, and morphologic evidence of lipodystrophy. Because the pathophysiological mechanism of HIV-associated lipodystrophy remains to be elucidated, we analyzed subcutaneous adipose tissues from the patients.

**Methods:**

All six patients had previously been treated with older nucleoside analogue reverse-transcriptase inhibitors (NRTIs; stavudine, didanosine or zidovudine). Abdominal and inguinal subcutaneous fat samples were obtained from the HIV+ patients with hemophilia and HIV− healthy volunteers (n = 6 per group), and analyzed via DNA microarray, real-time PCR, flow cytometry and immunohistochemistry.

**Results:**

The time from initial NRTI treatment to collecting samples were 21.7 years in average. Cytometric analysis revealed infiltration of inflammatory M1 macrophages into HIV-infected adipose tissue and depletion of adipose-derived stem cells, possibly due to exhaustion following sustained adipocyte death. Genetic analysis revealed that adipose tissue from HIV+ group had increased immune activation, mitochondrial toxicity, chronic inflammation, progressive fibrosis and adipocyte dysfunction (e.g. insulin resistance, inhibited adipocyte differentiation and accelerated apoptosis). Of note, both triglyceride synthesis and lipolysis were inhibited in adipose tissue from patients with HIV.

**Conclusions:**

Our findings provide important insights into the pathogenesis of HIV-associated lipodystrophy, suggesting that fat redistribution may critically depend on adipocytes’ sensitivity to drug-induced mitochondrial toxicity, which may lead either to atrophy or metabolic complications.

**Supplementary Information:**

The online version contains supplementary material available at 10.1186/s12981-022-00432-9.

## Introduction

More than 30 years have passed since 1431 hemophilia patients in Japan were infected with human immunodeficiency virus type 1 (HIV-1) after receiving transfusions of contaminated, unheated blood products [1]. The survival rate for patients living with HIV has dramatically improved alongside recent advancements in antiretroviral therapy (ART) [2], however, some hemophilia patients infected with HIV still suffer from lipodystrophy which is HIV-associated adverse effects of ART. And unfortunately, the precise mechanisms behind the development of lipodystrophy remains unrevealed.

After the introduction of ART in the mid-1990s, HIV-associated lipodystrophy was quickly identified as a significant adverse effect. It includes morphological changes (peripheral lipoatrophy and central obesity) and metabolic changes (dyslipidemia, insulin resistance, and hyperglycemia), affecting up to 80% of patients [3, 4]. Facial lipoatrophy and other body changes can lead to low self-esteem and poor medication adherence [5], while the metabolic complications of lipodystrophy have been associated with an increased risk of cardiovascular disease [6]. Previous studies have shown that antiretroviral drugs, especially older nucleoside analogue reverse-transcriptase inhibitors (NRTIs), provoke lipodystrophy [7]. It has been demonstrated that NRTI-induced mitochondrial toxicity is a primary cause of lipoatrophy [8], and long-term use of older NRTIs is closely linked to the development of severe lipoatrophy [9].

Although older NRTIs are rarely used in the current ART regimens, we need to continue treatment of the hemophilia patients who now suffer from HIV-associated lipodystrophy, for instance, management of facial lipoatrophy by filler injection [10, 11] or autologous fat grafting [12, 13]. However, publicly available data including gene expression from HIV-infected hemophilia patients is still limited. Therefore, this study was designed to investigate genetic trends and differential protein expression in adipose tissues from Japanese hemophilia patients with HIV-1 and facial lipoatrophy who were treated with old NRTIs, to reveal new insights into the pathogenesis of HIV-associated lipodystrophy.

## Methods

### Participants

This study was approved by the ethics committee from the institutional review board of National Center for Global Health and Medicine (NCGM; Tokyo, Japan) (NCGM-G-001598–00). Patients were recruited from the AIDS Clinical Center of NCGM if they granted informed consent and met the following inclusion criteria; (1) were infected with HIV-1 after receiving unheated blood products to treat hemophilia, (2) received an ART regimen including stavudine, didanosine or zidovudine (old NRTIs), (3) had plasma viral loads below 200 HIV-RNA copies/mL, and (4) had lipodystrophy presenting clinically evident facial lipoatrophy. Six patients met these criteria and were included in this prospective, open-label study (2014–2018; clinical trial number: UMIN000020379). Additionally, six HIV-negative, healthy male volunteers were enrolled in this study to obtain reference control data after providing their informed consent.

### Clinical specimens of adipose tissue

The six patients with HIV-associated facial lipodystrophy underwent liposuction of subcutaneous abdominal fat to enable lipotransfer for facial contouring. The lipoaspirates were purified by centrifugation at 1200×*g* for 3 min, the supernatant was discarded and the remaining fat portion was used for cellular analyses. In addition, a 1-cm^3^ sample of subcutaneous inguinal fat was collected from each of the six patients for genetic and histologic analyses. Also, HIV-negative lipoaspirates and excised fatty tissue were collected from the abdominal fat and inguinal fat of the six healthy volunteers, respectively, after obtaining informed consent before the procedure.

### Stromal vascular fraction isolation and flow cytometry

The stromal vascular fraction (SVF) was isolated from aspirated abdominal fat as previously described [14]. Briefly, each tissue was washed and digested in phosphate-buffered saline containing 0.075% collagenase (Wako Pure Chemicals, Osaka, Japan) for 30 min at 37 °C in a shaking water bath. After centrifugation (800×*g* for 10 min) and resuspension of the cell pellets, the SVF was obtained by filtering the cell suspension through a series of 100-, 70-, and 40-μm meshes (Millipore, Burlington, MA). Cell counts and viability were measured with an automated cell counter (NucleoCounter NC-100, ChemoMetec, Allerod, Denmark). The SVF samples were examined by flow cytometry using monoclonal antibodies against: CD45-fluorescein isothiocyanate (FITC), CD31-phycoerythrin (PE), CD14-PE, CD34-PE-Cy7 and CD206-allophycocyanin (APC) (BD Biosciences, Franklin Lakes, NJ). Cells were incubated with each antibody for 30 min (dilution, 1:10) and analyzed using a multicolor flow cytometer (MACS-Quant, Miltenyi Biotec) (n = 6 per group). Control gates were set based on staining with a negative isotype control; no more than 0.1% of cells stained positive using the isotype controls.

### Immunohistochemistry

The excised subcutaneous inguinal fat was fixed with Zinc Fixative (BD Biosciences), paraffin-embedded, sectioned at 5 μm and immunostained with the following primary antibodies: guinea pig anti-perilipin (1:200; Progen, Heidelberg, Germany) to stain the cytoplasm of adipocytes, rat anti-MAC2 (1:200; Cedarlane Corp., Burlington, Ontario, Canada) to stain monocytes/macrophages, rabbit anti-CD206 (1:100; Santa Cruz Biotechnology, Dallas, TX) to stain M2 macrophages, goat anti-CD34 (1:100; Santa Cruz Biotechnology) to stain adipose-derived stem cells (ASCs), and rabbit anti-von Willebrand factor (vWF; Dako, Santa Clara, CA) to stain vascular endothelial cells (VECs). For double fluorescence staining, appropriate secondary antibodies were used at a dilution of 1:200. An isotype IgG was used as a negative control for each staining step. Nuclei were stained with Hoechst 33342 (1:200; Dojindo, Kumamoto, Japan). Stained slides were examined under a fluorescence microscope (Keyence, Tokyo, Japan). The numbers of M1 and M2 macrophages, ASCs and VECs were counted in at least four field images for each sample (n = 6 per group).

### Comparative microarray analysis

Microarray was performed to analyze relative gene expression in the inguinal fat of HIV-infected and healthy control patients (n = 2 per group). Immediately after harvest, inguinal fat was mechanically homogenized and dissolved with ISOGEN, an RNA extraction reagent (Nippon Gene, Tokyo, Japan). Total RNA was purified using the RNeasy Mini kit (Qiagen, Hilden, Germany) according to the manufacturer's directions. Synthesis of cDNA was performed using the StepOnePlus real-time PCR system (Thermo Fisher Scientific, Waltham, MA) and SuperScript reverse transcriptase (Invitrogen, Waltham, MA). Then, cDNA probes were labeled with Cy3 using a SureTag DNA Labeling kit (Agilent Technologies, Santa Clara, CA) and hybridized with the SurePrint G3 Human GE 8 × 60 k Microarray Ver3.0 (G4858A, Agilent Technologies). Microarrays were scanned using a G2505C microarray scanner and read using Feature Extraction Software (Agilent Technologies). Finally, gene expression was analyzed using GeneSpring GX Software, Version 14.9 (Agilent Technologies).

### Selected gene expression analysis

Total RNA was isolated from the inguinal fat of HIV-infected and normal patients using an RNeasy Mini kit, followed by reverse transcription (n = 6 per group). Quantitative real-time polymerase chain reaction (PCR) was performed using the StepOnePlus real-time PCR system with fast SYBR Green PCR master mix (Thermo Fisher Scientific); the primer sequences were mainly derived from our previous publication [15], as listed in Tables 1, 2. Expression levels were calculated by the comparative CT method relative to the mean of two common endogenous reference genes, ACTB and GAPDH [16, 17].

**Table 1:** Primer sequences used for real-time PCR

Gene	Primer sequence (5ʹ–3ʹ)	
ACTB	Forward:	ATTGGCAATGAGCGGTTC
	Reverse:	GGATGCCACAGGACTCCAT
GAPDH	Forward:	CATGTTCGTCATGGGTGTGAACCA
	Reverse:	AGTGATGGCATGGACTGTGGTCAT
Leptin	Forward:	TGCCTTCCAGAAACGTGATCC
	Reverse:	CTCTGTGGAGTAGCCTGAAGC
PPARγ	Forward:	CTGTTTGCCAAGCTGCTCCAGAAA
	Reverse:	AAGAAGGGAAATGTTGGCAGTGGC
Adiponectin	Forward:	TGCTGGGAGCTGTTCTACTG
	Reverse:	TACTCCGGTTTCACCGATGTC
HIF1α	Forward:	TTGGCAGCAACGACACAGAAACTG
	Reverse:	TTGAGTGCAGGGTCAGCACTACTT
TNF-α	Forward:	AGGACGAACATCCAACCTTCCCAA
	Reverse:	TTTGAGCCAGAAGAGGTTGAGGGT
PAI-1	Forward:	TCTGCCCTCACCAACATTCTGAGT
	Reverse:	ACATGTCGGTCATTCCCAGGTTCT
GLUT1	Forward:	ATCGTGGCCATCTTTGGCTTTGTG
	Reverse:	CTGGAAGCACATGCCCACAATGAA

ACTB*, β*-actin; GAPDH, glyceraldehyde-3-phosphate dehydrogenase; PPAR-γ, peroxisome proliferator-activated receptor-γ; HIF-1α, hypoxia-inducible factor-1α; TNF-α, tumor necrosis factor-α; PAI-1, plasminogen activator inhibitor-1; GLUT1, glucose transporter 1

The primers sequences were derived from previous publications [15, 16]

**Table 2:** Cytometric analyses of stromal vascular fraction extracted from subcutaneous abdominal fat in patients with and without HIV

	HIV+	HIV−	*p*-value
Adipose-derived stem cells (× 10^5^/ml)	0.96 ± 0.20	1.39 ± 0.22	0.043*
Vascular endothelial cells (× 10^4^/ml)	9.31 ± 1.99	6.69 ± 1.53	0.113
Hematopoietic cells (× 10^5^/ml)	1.24 ± 0.28	1.11 ± 0.17	0.712
Other cells ^a^ (× 10^4^/ml)	6.67 ± 1.79	5.05 ± 1.06	0.103
M1 macrophages (%)^b^	16.1 ± 4.24	7.08 ± 1.81	0.014*
M2 macrophages (%)^b^	42.3 ± 8.53	53.5 ± 5.19	0.088
Total macrophages (× 10^4^/ml)	4.20 ± 1.09	3.06 ± 0.98	0.164
CD14^+^/CD45^−^/CD34^+^ cells ^c^ (× 10^4^/ml)	6.74 ± 0.88	0	0*

^*^ Statistically significant

^a^ Mural cells, fibroblasts, smooth muscle cells and others

^b^ Relative composition of macrophage phenotypes to CD45 + cells

^c^ Possibly derived from bone marrow-derived stem cells

### Statistics

Data were analyzed using SPSS package 23.0 (SPSS, Inc., Chicago, IL) and Kyplot 2.0 (Freeware). Based on the Kolmogorov/Smirnov test, our data were normally distributed within the donor population. Therefore, Welch’s t-test was used to assess differences between the two groups. A threshold of *p* < 0.05 was considered statistically significant, unless otherwise stated.

## Results

### Cellular analysis

All study participants were Japanese males with an average age of 42.7 ± 6.0 (mean ± SD) in the HIV+ group and 44.1 ± 8.3 in the control group. The history of old NRTIs history of the patients are delineated in Additional file 1 Table S1. The time which has elapsed since the patients received NRTI associated with lipodystrophy were 21.7 years in average (ranged from 19.4 to 26.5 years). None of the patients have currently been administrated neither stavudine, didanosine nor zidovudine. The SVF cells isolated from the abdominal fat of HIV patients and healthy controls were analyzed by flow cytometry. The total cell count and viability of the SVF were 4.2 ± 0.9 × 10^5^/ml and 89.8 ± 4.3% in the HIV+ group and 3.9 ± 0.5 × 10^5^/ml and 93.8 ± 3.1% in the healthy control group, respectively. There was no significant difference in cell number, viability and viable cell number between the two groups.

**Fig. 1:**
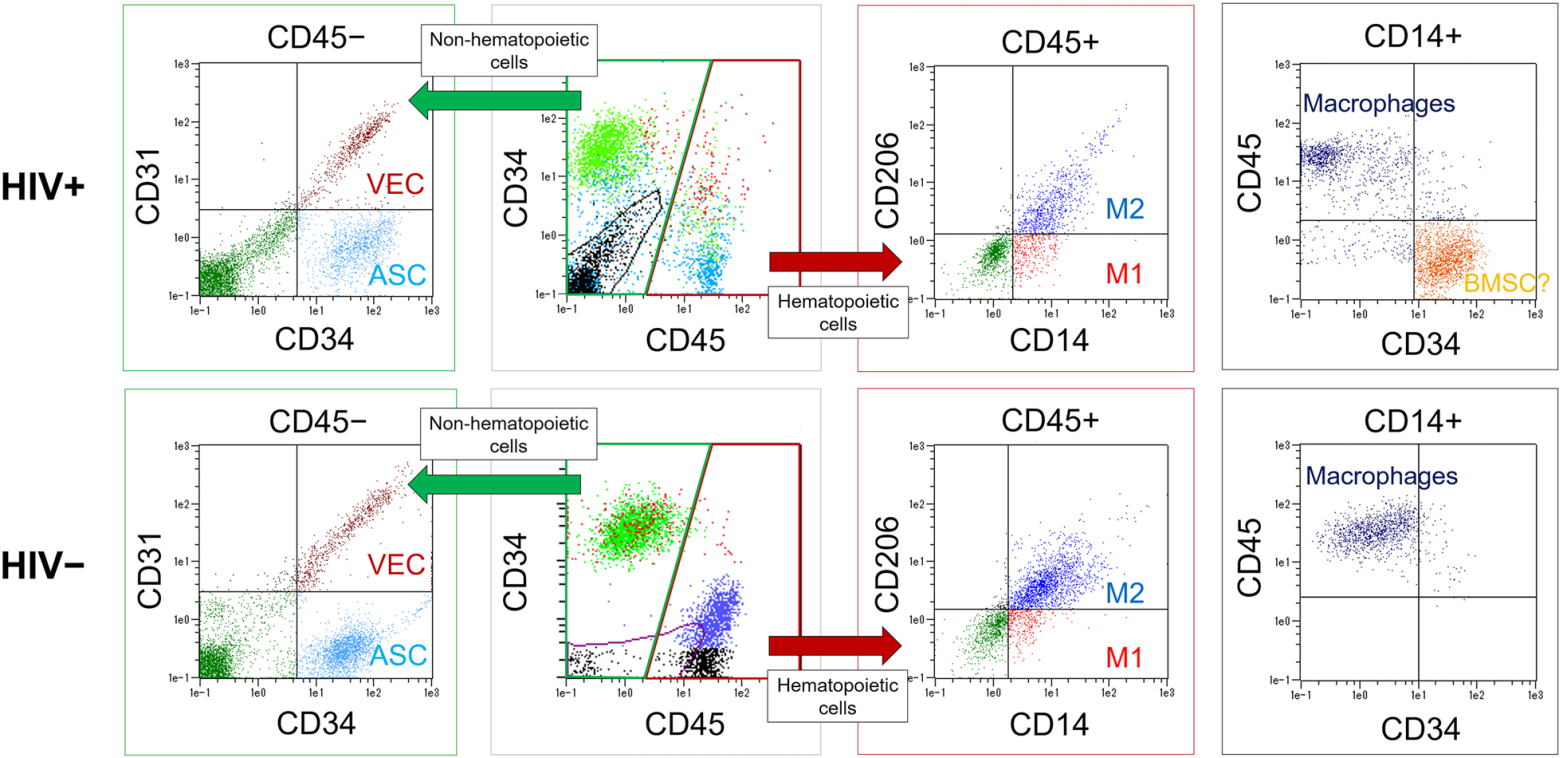
Representative plots of flow cytometry data of stromal vascular fraction extracted from subcutaneous abdominal fat. Adipose-derived stem cells (ASCs) are CD45^−^/CD31^−^/CD34^+^, vascular endothelial cells (VECs), hematopoietic cells (HCs) are CD45^+^, M1 macrophages are CD45^+^/CD14^+^/CD206^−^, and M2 macrophages are CD45^+^/CD14^+^/CD206^+^. CD14^+^/CD45^−^/34^+^ cells in the HIV + group may be bone marrow-derived stem cells (BMSCs) as described in “Discussion” section

Cells from the SVF were characterized on the basis of multiple cell surface antigens. First, a significantly lower number (*p* = 0.043) and relative proportion (*p* = 0.020) of CD45^−^/CD31^−^/CD34^+^ ASCs was found in the HIV+ group (1.0 × 10^5^; 23.8%) compared with the control group (1.4 × 10^5^; 36.3%) (Fig. 1A). No significant difference was detected in the number of CD45^−^/CD31^+^/CD34^−^ VECs, hematopoietic cells or other cells between the two groups, although the HIV+ group had a slightly higher number of VECs (0.9 × 10^5^) than the control group (0.7 × 10^5^) (*p* = 0.113). Second, we evaluated the relative frequency of two types of macrophages: CD45^+^/CD14^+^/CD206^−^ inflammatory macrophages (M1) and CD45^+^/CD14^+^/CD206^+^ anti-inflammatory macrophages (M2). A comparison of the relative frequency of M1 and M2 cells among the CD45^+^ cell subset revealed that fat from HIV-infected patients had significantly more M1 cells compared with healthy samples (16.1% vs. 7.1%, *p* = 0.014); however, there was no significant difference in the frequency of M2 cells (42.3% vs. 53.5%, *p* = 0.088) or in the total number of macrophages between the two groups (7.3 × 10^4^ vs. 6.8 × 10^4^) (Fig. 1B). Moreover, CD14^+^ cells were analyzed based on patterns of CD45 and CD34 expression. Whereas healthy control subjects contained only CD45^+^/CD14^+^/CD34^−^ macrophages, HIV patients showed CD45^−^/CD14^+^/CD34^+^ cells, which made up 42.1% of the CD14^+^ cell population (Fig. 1C). Representative flow cytometry plots are shown in Fig. 1D.

### Immunohistochemistry

**Fig. 2:**
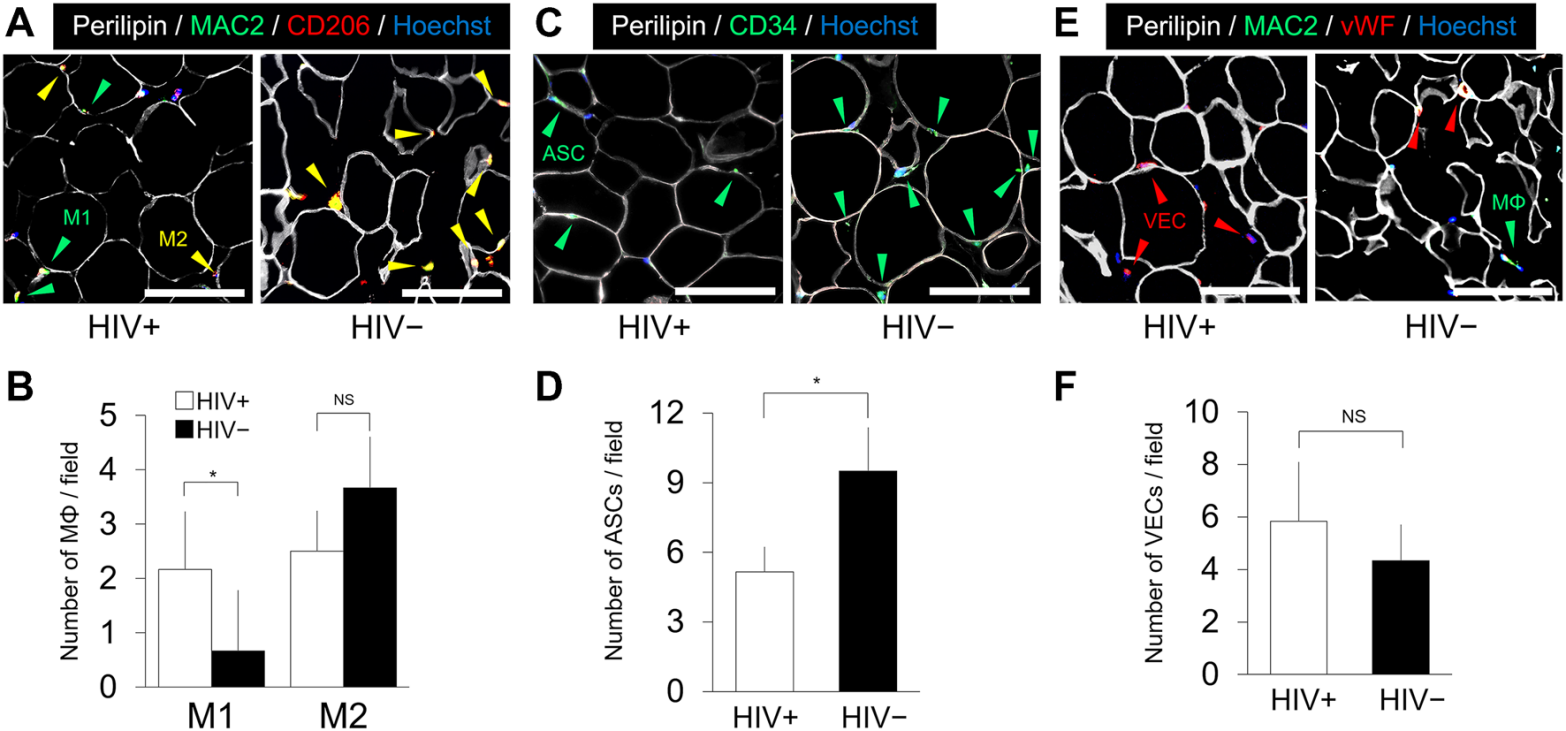
**A** Immunohistology for adipocytes (perilipin) and macrophages (MAC2 and CD206) of subcutaneous inguinal adipose tissue. MAC2^+^/CD206^−^ macrophages (M1) were observed in fat from HIV patients (*green arrows*), while the greater part of macrophages were MAC2^+^/CD206^+^ (M2) in normal fat (*yellow arrows*). Scale bars = 100 μm. **B** Number of M1 and M2 per field. *p = 0.030. **C** Immunohistology for adipose stem cells (ASCs) of subcutaneous inguinal adipose tissue. Fat from patients with HIV had fewer ASCs compared to healthy controls (*green arrows*). Scale bars = 100 μm. **D** Number of ASCs per field. *p = 0.002. **E** Immunohistology for vascular endothelial cells (VECs) of subcutaneous inguinal adipose tissue. VECs were more abundant in fat from patients with HIV. vWF; von Willebrand factor. MΦ; macrophage. Scale bars = 100 μm. **F** Number of VECs per field. The number of VECs did not differ significantly

Microsections of inguinal adipose tissues were immunostained to show the cytoplasm of adipocytes (perilipin), macrophages (MAC2), M2 macrophages (CD206), ASCs (CD34), VECs (vWF), and nuclei (Hoechst). The size and shape of adipocytes were similar between the two groups. Whereas most macrophages in the fat from healthy controls were M2 cells, fat from patients with HIV had a significantly larger number of M1 cells (*p* = 0.030), suggesting chronic inflammation (Fig. 2A, B). Although ASCs were observed in samples from both groups, fat samples from patients with HIV contain significantly fewer ASCs (*p* = 0.002), suggesting its impaired adipogenic potential (Fig. 2C, D). With regard to vessel density, although VECs were more abundant in some samples from patients with HIV, the difference was not statistically significant (*p* = 0.120) (Fig. 2E, F).

### Comparative gene expression analysis

**Fig. 3:**
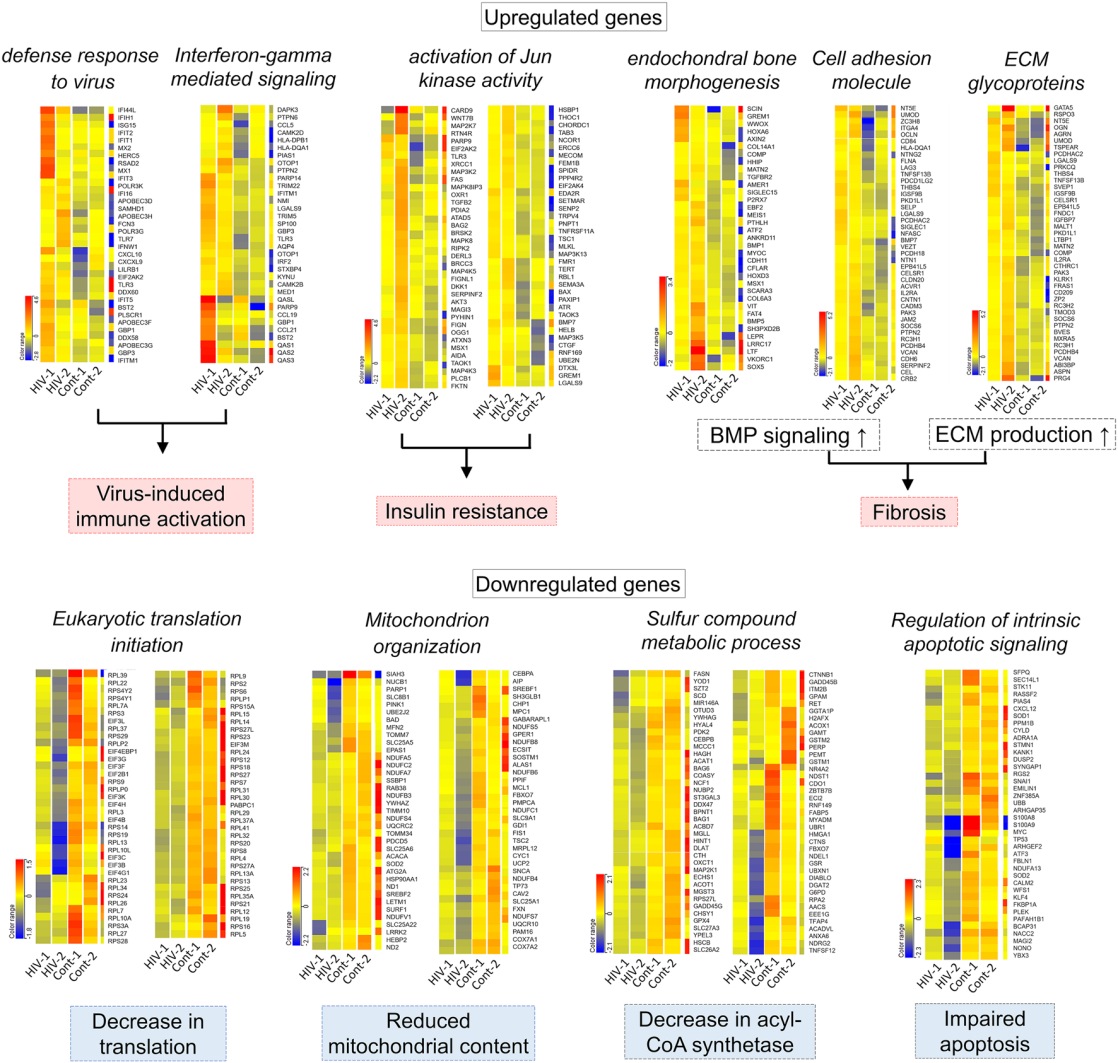
Microarray analyses of excised fat from the inguinal region. Heat maps show upregulated and downregulated gene ontologies

Microarray was performed to identify differences in the gene expression profiles of adipose tissue from patients with HIV and healthy controls. Individual analyses were performed on the 9,604 genes that were either 1.5-fold upregulated or downregulated (t test *p* < 0.05; 802 entities). Gene ontology analyses of the two populations indicated distinct characteristic ontologies that are summarized in Additional file 2: Fig. S1 and visualized by heat mapping in Fig. 3 and Additional file 3: Fig. S2. Patients with HIV had significant upregulation of genes related to immune activation, insulin resistance and fibrosis, and significant downregulation of genes involved in mitochondrial organization, acyl-CoA synthetase and regulation of apoptosis. Expression of several genes related to mitochondrial function, lipid metabolism and adipogenesis, represented by the corrected intensities of duplicate spots, were shown in Table 3.

**Table 3:** Comparison of gene expression data extracted by microarray

Gene	HIV+	HIV–	HIV+/HIV–
Mitochondrial energy metabolism			
*COX3*	0.77	1.33	0.58
Mitochondrial biogenesis			
*POLG1*	0.81	1.00	0.81
Lipid metabolism			
*FASN*	0.57	1.39	0.41
Adipogenesis			
*CEBPB*	0.69	1.34	0.51
*SREBF1*	0.88	1.41	0.63
*PPARG*	0.84	1.12	0.75
*LMNA*	0.76	1.18	0.64
*KLF15*	0.52	1.25	0.42
Apoptosis			
*FAS*	1.87	0.73	2.56
*TNFAIP3*	1.29	0.96	1.35
*SOD1*	1.29	1.00	1.29

The values shown are the average of individual intensities of duplicate spots

*COX3,* cytochrome c oxidase subunit III; *POLG1*, polymerase γ (catalytic subunit); *FASN*, fatty acid synthase; *CEBPB*, CCAAT/enhancer binding protein β; *SREBF1*, sterol regulatory element-binding transcription factor 1; *PPARG*, peroxisome proliferator-activated receptor gamma; *LMNA*, Lamin A; *KLF15*, kruppel-like transcription factor 15; *FAS*, factor of apoptotic stimulus; *TNFA*, tumor necrosis factor alfa; *SOD1*, increase of superoxide dismutase 1

Analysis using the Kyoto Encyclopedia of Genes and Genomes (KEGG) database revealed that fat from patients with HIV had gene expression patterns consistent with inhibited insulin signaling and decreased synthesis of triglycerides (Additional file 4: Fig. S3). Additionally, genes encoding elements downstream of peroxisome proliferator-activated receptor-γ (*PPAR-γ*) such as *FABP4*, *perilipin* and *AQP7* were downregulated, suggesting inhibited differentiation, maturation and survival of adipocytes as well as reduced fatty acid uptake. Finally, despite having enhanced cAMP signaling, patients with HIV had reduced expression of genes related to lipolysis. All microarray data obtained from our study were deposited with the National Center for Biotechnology Information Gene Expression Omnibus Database (accession no. GSE147162).

### Selected gene expression analysis

**Fig. 4:**
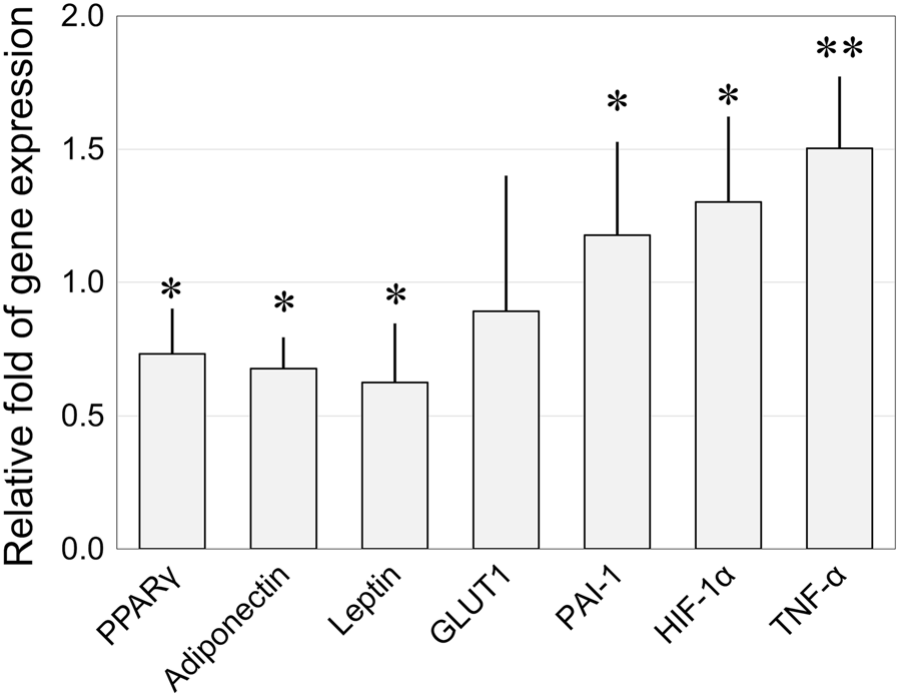
Transcriptional profiles of inguinal adipose tissues of patients with HIV-1 compared to healthy controls, quantified by real-time PCR. There was a decrease in key regulatory proteins of adipocytes including PPAR-γ (peroxisome proliferator-activated receptor), adiponectin and leptin and an increase in inflammation-associated genes including PAI (plasminogen activator inhibitor)-1, HIF (hypoxia-inducible factor)-1α and TNF-α (tumor necrosis factor). GLUT1; glucose transporter 1. * *p* < 0.05, ** *p* < 0.01

The relative expression of several genes in the inguinal fat was confirmed via real-time PCR (Fig. 4). Patients with HIV had significantly decreased expression of key regulatory proteins in adipocytes, PPAR-γ, adiponectin and leptin, relative to the healthy controls (*p* < 0.05 in all). An analysis of obesity-related genes revealed that patients with HIV had significantly higher expression of PAI-1 and HIF-1α (*p* < 0.05 in both) and not significantly different expression of GLUT1. Expression of TNF*-*α, an inflammatory cytokine, was greatly upregulated in patients with HIV (*p* = 0.009).

## Discussion

Although older NRTIs which mostly induce lipodystrophy are rarely used in the current clinical practice, the patients who had been treated with such NRTIs continue to live with lipodystrophy. Therefore, there is still a need for elucidation of the pathologic mechanism of HIV-lipodystrophy, to actualize better treatment of those patients. Moreover, as many investigators have previously reported, HIV-associated lipodystrophy is the result of a complex interplay between the HIV-1 virus, toxic side effects of antiretroviral drugs and host characteristics [18]. Subtle changes in fat quantity and metabolism were observed in patients with HIV prior to the use of antiretroviral drugs [19], in patients with other infections (e.g., tuberculosis) [20] and in patients with non-infectious proinflammatory conditions (e.g., malignancy-induced cachexia) [21]. Thus, considering the pathogenesis of HIV-associated lipodystrophy from the perspective of an immune-metabolic imbalance seems to be useful for further understanding of various diseases presenting lipodystrophic condition.

### Adipose-derived stem cells in HIV-associated lipodystrophy

We compared the cellular composition of SVF isolated from the abdominal fat of lipodystrophic patients with HIV and healthy controls using flow cytometry. First, we focused on ASCs which play a central role in the regeneration of adipose tissue, by differentiation into adipocytes and release of angiogenic factors [22]. There were fewer ASCs in fat from patients with HIV compared to healthy patients. This decrease in ASC quantity was also confirmed by immunohistochemistry. We believe this is due to the exhaustion of ASCs in the process of sustained and progressive lipoatrophy. On the other hand, there was no significant difference in the number of VECs, hematopoietic cells, or total SVF cells in patients with HIV. Though not a statistically significant difference, patients with HIV had a higher average number of VECs (*p* = 0.113), in good agreement with several previous reports [23].

### Macrophages in HIV-associated lipodystrophy

Macrophages are a key modulator of the immune response to pathogens. In this work, we measured inflammatory macrophages (M1) and anti-inflammatory macrophages (M2), which are typically regarded as the two extremes in a continuum of functional macrophage phenotypes [24]. We found that fat from lipodystrophic patients with HIV contained a significantly greater number of M1 relative to healthy controls, whereas there was no significant difference in M2 or in total number of macrophages between the two groups. Because monocytes and macrophages expressing CD14 are more susceptible to HIV infection, the host immune response to viral infection rather than the drug toxicity is of particular importance in this shift of macrophages toward M1 phenotype [25]. Increased infiltration of M1 macrophages leads to the production of proinflammatory cytokines that result in inflammation and functional impairment of adipocytes [26].

Another interesting finding in this study was the presence of CD45^−^/CD14^+^/CD34^+^ cells in fat from patients with HIV (comprising 42.1% of all CD14^+^ cells), which were not observed in healthy controls. Usually, CD45^+^ hematopoietic stem cells differentiate into CD14^+^ monocytes in the bone marrow. However, previous studies have reported the isolation of CD45^−^/CD14^+^/CD34^+^ low-adherent multipotent mesenchymal cells from the bone marrow [27]. In addition, after HIV-infected monocytes/macrophages extravasate from the bloodstream to tissues, they reside for a longer period of time than non-infected cells [28], and long-term residence in adipose tissue decreases CD45 expression of the cells [14, 27]. Actually, we reported a subpopulation of CD45^+^/CD14^+^/CD34^+^ macrophages localized in the perivascular region of adipose tissue in healthy patients, which were capable of differentiating into multiple mesenchymal lineages and lost expression of CD45 by passage 3 resulting in an expression profile similar to ASCs [29]. Thus, we hypothesize that the CD45^−^/CD14^+^/CD34^+^ cells found in this study are bone marrow-derived stem cells that migrated to HIV-infected fat in compensatory response to ASC deficiency and lipoatrophy, however, additional work is required to address the specific origin of this cell population.

### Inflammation and fibrosis in HIV-associated lipodystrophy

We previously found that more than 90% of macrophages that reside in normal adipose tissue are M2 phenotype [29]. While adipose tissue-resident M2 cells are involved in tissue remodeling and anti-inflammatory reactions [30], M1 cells cause inflammation and impair adipocyte function leading to insulin resistance and dyslipidemia, which was also confirmed in HIV-infected lipoatrophic patients in previous reports [26, 31]. The qualitative change in macrophage phenotype from M2 into M1 is typically observed in obesity, where adipocyte hypertrophy is accompanied by higher numbers of macrophages and a greater polarization toward proinflammatory M1 cytokine phenotypes [32]. Prior evidence of macrophage activation and inflammation in the subcutaneous fat of individuals with insulin resistance implies the mechanistic similarity between HIV-associated lipodystrophy and metabolic disorders [33].

Comparative gene analyses of fat samples from patients with and without HIV produced data that mirrored our flow cytometric findings. Genes related to host immune responses and inflammation were upregulated in the HIV+ group, suggesting that activated M1 [26] and CD4^+^ T cells [34] were potential sources of proinflammatory cytokine production. Besides, expression of genes responsible for extracellular matrix production was upregulated in the HIV+ group, suggesting progressive fibrosis. Fibrosis, which was also described in the context of other organs such as the liver and myocardium in HIV patients, indicates a proinflammatory and profibrotic state of adipose tissue [35].

### Adipocyte dysfunction by mitochondrial toxicity

Since the introduction of NRTIs in the mid-1990s, mitochondrial toxicity has been documented as the most severe form of ART-induced adipocyte toxicity. Adipocyte mitochondria plays a central role in metabolism, energy expenditure and clearance of reactive oxygen species. During adipocyte differentiation, mitochondrial activity dramatically increases to promote adipogenesis and maturation into adipocytes [36]. Thus, mitochondrial dysfunction in adipocytes is often accompanied by impaired fatty acid metabolism, disturbed oxidative phosphorylation, altered adipokine secretion and reduced glucose uptake [37], resulting in inhibited adipogenesis and activated apoptosis.

Our study revealed decreased expression of several genes involved in mitochondrial function in fat from HIV patients, including inhibited expression of *POLG1* which was suggested as one of key factors in NRTI-induced lipoatrophy [8]. Relatedly, we found that transcription factors important to adipogenesis such as *SREBF1* [38], *CEBPB* [39], and *PPARG* [40, 41] were downregulated in patients with HIV in accordance with previous studies [17, 26, 41]. Oxidative stress due to mitochondrial toxicity, indicated by an upregulation of *SOD1*, also causes inflammation and accelerates apoptosis [42]. In particular, our finding of remarkably increased TNF-α (which is associated with inflammation and apoptosis) in HIV patients is in good agreement with prior studies [42]. Key regulatory genes of apoptosis such as *Fas* were also upregulated in patients with HIV in this study. All these findings reflect impairment of adipocyte differentiation and function, due to mitochondrial toxicity.

### Changes in lipid metabolism and fat distribution

Fat redistribution in HIV-infected patients are considered consequence of metabolic complications including toxicity of old NRTIs. According to previous reports, HIV patients with lipodystrophy present with a higher index of total lipolysis [43] and increased circulating free fatty acids [17, 43, 44] compared to HIV-negative controls. In our study, however, KEGG pathway analysis revealed a lower index of lipolysis in HIV patients. Nevertheless, inhibited synthesis of triglycerides and reduced intake of free fatty acid causes elevated free fatty acids [45], which induces inflammatory M1 migration, decreases adiponectin production and leads to insulin resistance in adipocytes [43].

Our results may help elucidate the etiology of fat redistribution in patients with HIV. The accumulation of visceral fat after HIV infection is often accompanied by systemic inflammation, insulin resistance, dyslipidemia and an increased risk of cardiovascular diseases [46, 47]; very similar to the profile of patients with metabolic syndrome. Although the acute phase of inflammation accelerates lipolysis, chronic inflammation causes insulin resistance which is strongly associated with dysregulated lipolysis and dyslipidemia (increased triglycerides). Of interest, previous reports have suggested that visceral adipocytes are less susceptible to the toxicity of older NRTIs relative to subcutaneous adipocytes, as measured by decreases in gene expression of *PPAR-γ* [48–50] and so on, potentially due to their higher mitochondrial content. Thus, visceral fat, which retains most of its mitochondrial function despite NRTI treatment, may expand in the presence of an inflammatory environment and impaired insulin-stimulated glucose uptake resulting in central obesity. On the contrary, subcutaneous fat, which is sensitive to NRTI toxicity, is more severely damaged resulting in peripheral lipoatrophy. Therefore, we hypothesize that identical inflammatory conditions cause opposite effects for different types of fat, depending on their sensitivity to mitochondrial toxicity of NRTIs. Previous studies showed that stopping or replacing older NRTI medications often leads to improvements in limb fat atrophy but not in visceral fat accumulation [51, 52], which may support our hypothesis because metabolic changes are hard to restore compared to direct adverse drug reactions.

This study has several limitations. This is a very small case series. We carefully analyzed abdominal and inguinal subcutaneous adipose tissues, however, collecting samples from the same location would be more desirable approach. Also, these sites were the areas usually affected by lipohypertrophy [53], and additional analyses of the areas severely affected by lipoatrophy (such as face and limbs) might be helpful for further understanding of pathology of HIV-associated lipodystrophy. Finally, The pathogenesis of lipodystrophy we provided is inferential, and additional researches are required to validate its reliability.

## Supplementary Information

**Additional file 1: Table S1.** The history of antiretroviral therapy of the patients with HIV-associated lipodystrophy.

**Additional file 2: Figure S1. ** Summary of the functional categories of genes differentially expressed in adipose tissue from lipodystrophic patients with HIV.

**Additional file 3: Figure S2.** Heat maps of upregulated and downregulated gene ontologies.

**Additional file 4: Figure S4.** KEGG pathway analysis suggested inhibited insulin signaling, reduced adipocyte differentiation and function, and decreased synthesis of triglycerides and lipolysis in the subcutaneous far fromHIV+ group.

## Data Availability

The datasets generated and/or analyzed during the current study are not publicly available due to ethical and legal reasons, but are available from the corresponding author on reasonable request.
