# Supplementary material for: Genetic and cytometric analyses of subcutaneous adipose tissue in patients with hemophilia and HIV-associated lipodystrophy
Source: AIDS Res Ther. 2022 Mar 4;19:14. doi: 10.1186/s12981-022-00432-9 (PMC8895510; doi:10.1186/s12981-022-00432-9)
Supplement: Supplementary file 1 — Additional file 1: Table S1. The history of antiretroviral therapy of the patients with HIV-associated lipodystrophy. [file 12981_2022_432_MOESM1_ESM.docx]

**Supplementary Table 1. The history of antiretroviral therapy of the patients with HIV-associated lipodystrophy**

| Case | Age (yr) | Sex | ART history | d4T exposure (months) | ddI exposure (months) | AZT exposure (months) |
| --- | --- | --- | --- | --- | --- | --- |
| 1 | 45 | Male | 1995/04 **AZT**+**ddI** 1997/07 NFV+**d4T**+3TC 2002/05 LPV/rtv+EFV+ABC 2002/05 LPV/rtv+TDF+ABC 2008/11 RAL+TDF+ABC | 58 | 27 | 27 |
| 2 | 43 | Male | 1989/12 **AZT** 1991/09 **AZT**+**ddI** 1996/10 **AZT**+ddC 1996/11 SQV＋**AZT** 1997/08 IDV+**AZT**+3TC (for 7 days) 1997/08 NFV+**AZT**+3TC 1998/03 SQV/RTV+**d4T**+**ddI** 2000/02 APV+EFV+ABC (for 9 days) 2000/02 APV+NVP+ABC 2001/01 LPV/rtv+ABC+3TC 2009/01 RAL+TDF/FTC 2009/05 DRV/rtv+TDF/FTC 2014/09 DTG+TDF/FTC | 23 | 72 | 99 |
| 3 | 51 | Male | 1994/01 **AZT** (for 1 week, interruption) 1995/07 **d4T**+**ddI** 1997/08 IDV+**d4T**+3TC 1999/11 IDV/RTV+**d4T**+3TC 2001/03 EFV+**d4T**+3TC 2002/04 EFV+ABC+3TC 2004/12 (interruption: SMART) 2005/05 EFV+ABC/3TC 2008/05 ATV/rtv+ABC/3TC 2008/12 DRV/rtv+RAL+ABC/3TC 2012/09 DRV/rtv+RAL 2017/05 RPV+RAL 2017/07 DRV/rtv+DTG | 80 | 25 | 0 |
| 4 | 38 | Male | 1991/02 **AZT** 1995/08 **AZT**+**ddI** 1996/07 **AZT**+ddC 1996/09 IDV+**AZT**+ddC 1999/05 NFV+**d4T**+3TC 1999/10 RTV/SQV+EFV 2004/02 ATV/rtv+EFV 2006/09 ATV/rtv+TDF/FTC 2007/04 ATV/rtv+TDF (half dose)+3TC 2007/05 ATV/rtv+EFV 2008/09 FPV+EFV+RAL (for 9 days) 2008/09 ATV/rtv+EFV 2009/02 ATV/rtv+EFV+3TC 2009/03 ATV/rtv+ETR+3TC 2009/05 ETR+RAL+3TC 2012/08 EFV+RAL+3TC 2015/09 RPV+DTG+3TC | 5 | 11 | 99 |
| 5 | 32 | Male | 1996/07 **AZT**+ddC 1996/08 IDV+**AZT**+ddC 1997/09 NFV+**d4T**+3TC 1998/02 SQV/RTV+**d4T**+3TC 2012/03 RAL+ABC/3TC 2017/02 DTG+ABC/3TC | 174 | 0 | 14 |
| 6 | 43 | Male | 1996/08 **AZT**+3TC 1997/09 **AZT**+3TC+NVP 1997/12 **AZT**+3TC 1998/01 IDV+**d4T**+3TC 2000/01 IDV/RTV+**d4T**+3TC 2001/08 EFV+**d4T**+3TC 2001/09 ABC+**d4T**+3TC 2002/11 3TC+ABC+TDF 2003/12 ABC+**d4T**+3TC 2005/10 (interruption) 2006/12 FPV/rtv+ABC/3TC 2010/11 DRV/rtv+ABC/3TC 2014/07 DTG+ABC/3TC | 79 | 0 | 17 |

ART, antiretroviral therapy; d4T, stavudine; ddI, didanosine; AZT, zidovudine
