# Supplementary figures and images for: Genetic and cytometric analyses of subcutaneous adipose tissue in patients with hemophilia and HIV-associated lipodystrophy
Source: AIDS Res Ther. 2022 Mar 4;19:14. doi: 10.1186/s12981-022-00432-9 (PMC8895510; doi:10.1186/s12981-022-00432-9)

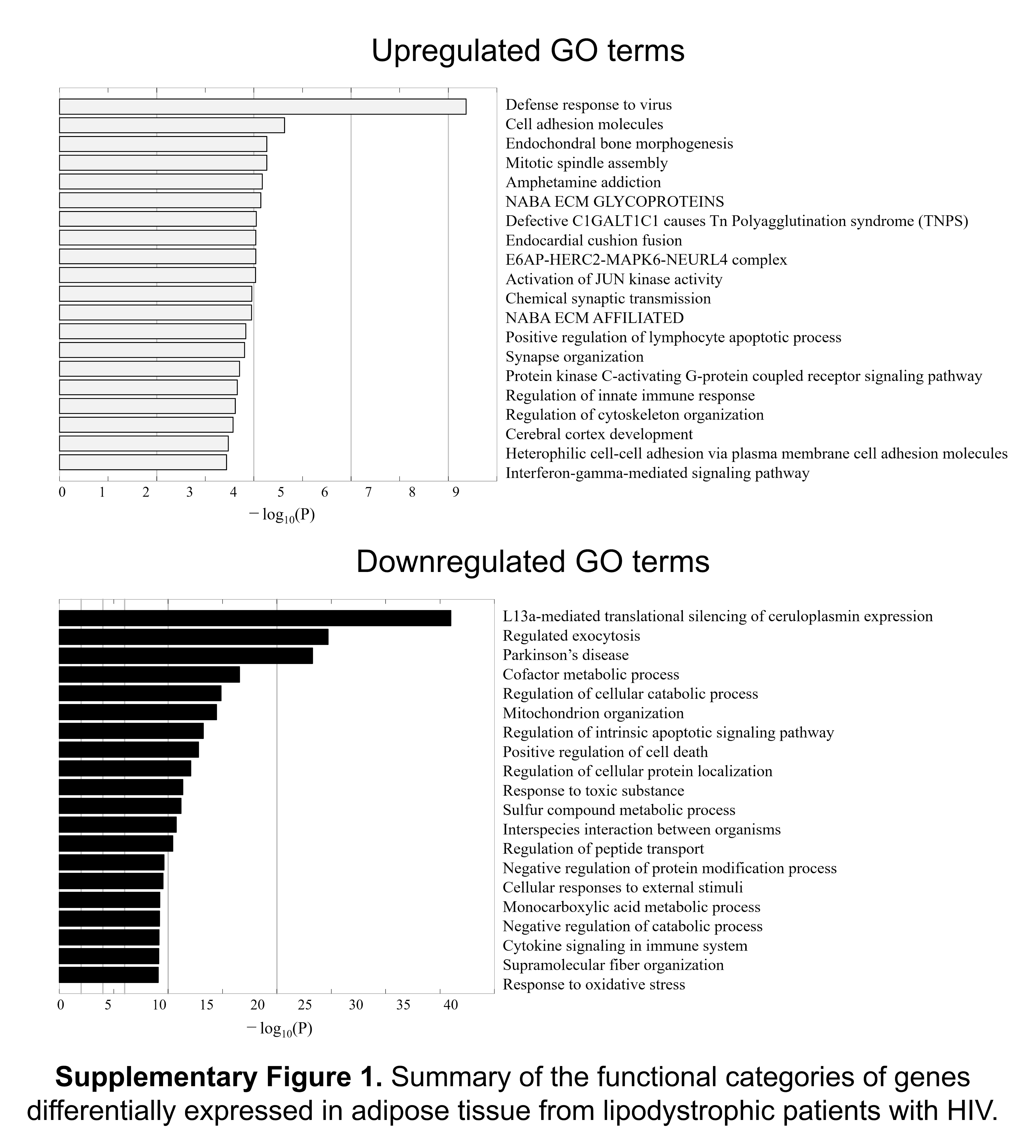

Supplement: Supplementary file 2 — Additional file 2: Figure S1. Summary of the functional categories of genes differentially expressed in adipose tissue from lipodystrophic patients with HIV. [file 12981_2022_432_MOESM2_ESM.tif]

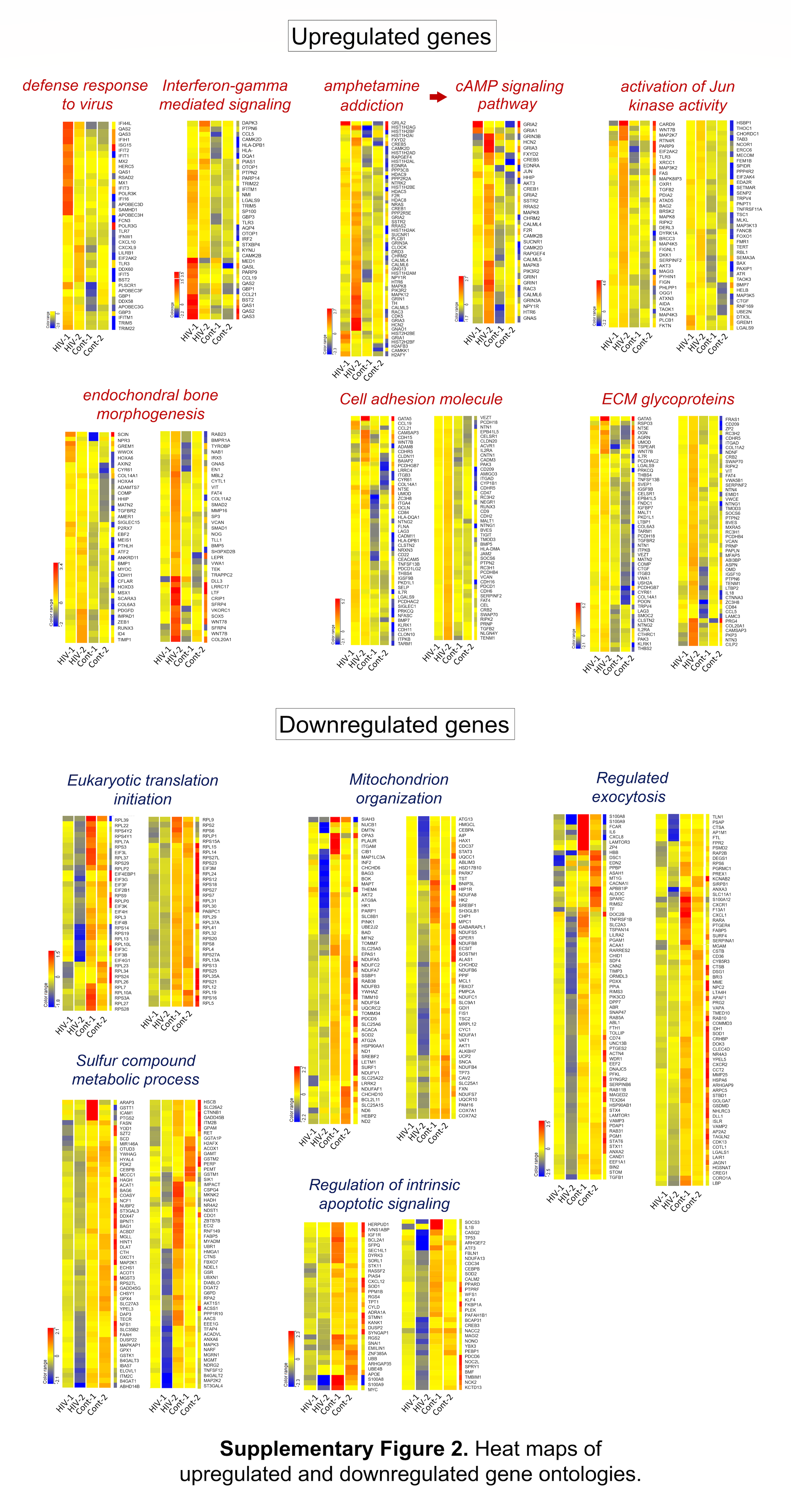

Supplement: Supplementary file 3 — Additional file 3: Figure S2. Heat maps of upregulated and downregulated gene ontologies. [file 12981_2022_432_MOESM3_ESM.tif]

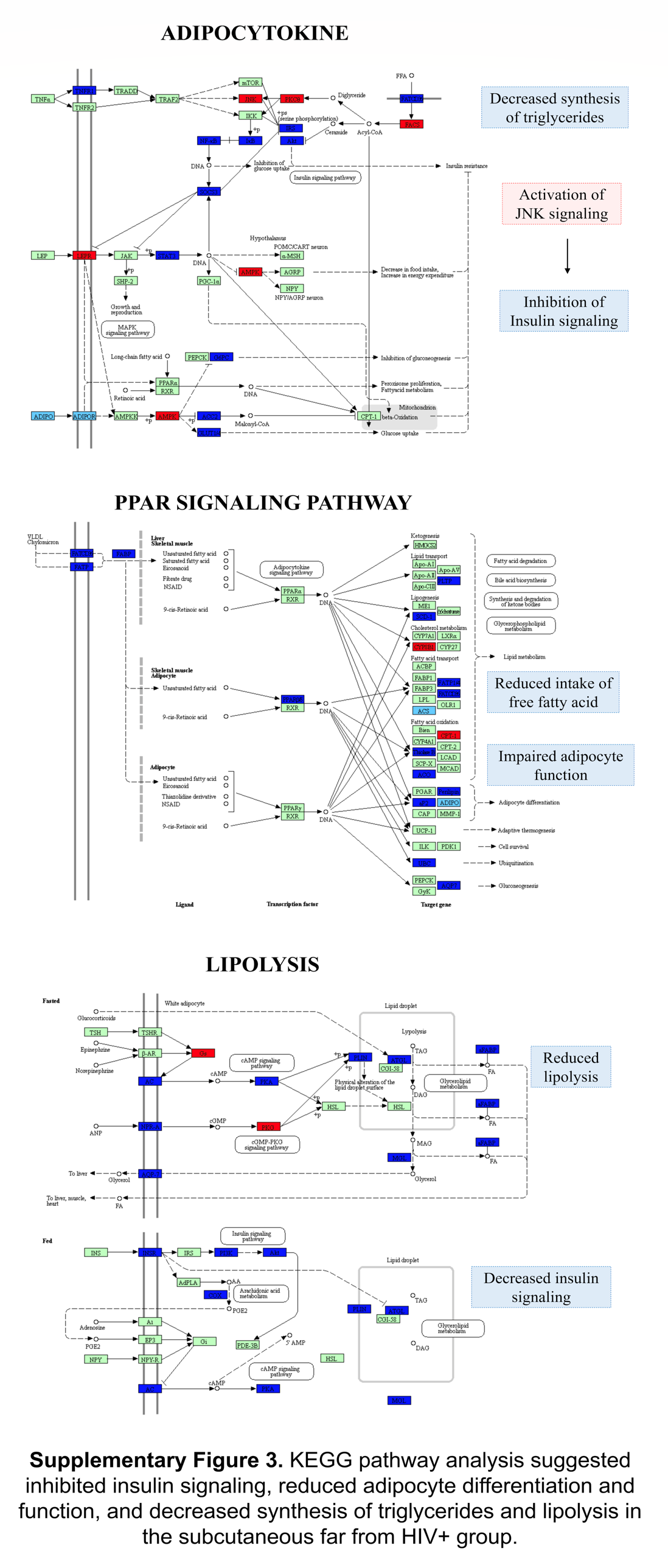

Supplement: Supplementary file 4 — Additional file 4: Figure S4. KEGG pathway analysis suggested inhibited insulin signaling, reduced adipocyte differentiation and function, and decreased synthesis of triglycerides and lipolysis in the subcutaneous far fromHIV+ group. [file 12981_2022_432_MOESM4_ESM.tif]
